# Supplementary material for: Modeling glioblastoma heterogeneity as a dynamic network of cell states
Source: Mol Syst Biol. 2021 Sep 16;17(9):e10105. doi: 10.15252/msb.202010105 (PMC8444284; doi:10.15252/msb.202010105)
Supplement: Supplementary file 5 — Source Data for Figure 3 [file MSB-17-e10105-s001.zip › Figure3A_sourcedata/GSEA_3065/nosigdb_state3.GseaPreranked.1622718426028/RICHARDS_NEFTEL_CELL_2019_MES1.html]

Details for gene set RICHARDS\_NEFTEL\_CELL\_2019\_MES1[GSEA]

|  || Dataset | state3 |
| Phenotype | NoPhenotypeAvailable |
| Upregulated in class | na\_neg |
| GeneSet | RICHARDS\_NEFTEL\_CELL\_2019\_MES1 |
| Enrichment Score (ES) | -0.5255274 |
| Normalized Enrichment Score (NES) | -1.5683646 |
| Nominal p-value | 0.015050167 |
| FDR q-value | 0.033945918 |
| FWER p-Value | 0.592 |
Table: GSEA Results Summary

  

Fig 1: Enrichment plot: RICHARDS\_NEFTEL\_CELL\_2019\_MES1      
 Profile of the Running ES Score & Positions of GeneSet Members on the Rank Ordered List

  

| PROBE | GENE SYMBOL | GENE\_TITLE | RANK IN GENE LIST | RANK METRIC SCORE | RUNNING ES | CORE ENRICHMENT || 1 | TIMP1 |  |  | 35 | 0.470 | 0.0634 | No |
| 2 | EMP3 |  |  | 91 | 0.354 | 0.1079 | No |
| 3 | CTSB |  |  | 392 | 0.219 | 0.1050 | No |
| 4 | A2M |  |  | 585 | 0.188 | 0.1100 | No |
| 5 | FN1 |  |  | 973 | 0.140 | 0.0857 | No |
| 6 | ACTN1 |  |  | 1079 | 0.132 | 0.0927 | No |
| 7 | GJA1 |  |  | 1160 | 0.125 | 0.1015 | No |
| 8 | SPP1 |  |  | 1358 | 0.111 | 0.0948 | No |
| 9 | EMP1 |  |  | 1624 | 0.096 | 0.0782 | No |
| 10 | CD44 |  |  | 1748 | 0.089 | 0.0768 | No |
| 11 | NPC2 |  |  | 1842 | 0.084 | 0.0782 | No |
| 12 | EFEMP1 |  |  | 2265 | 0.066 | 0.0392 | No |
| 13 | C1R |  |  | 2407 | 0.060 | 0.0317 | No |
| 14 | NAMPT |  |  | 2583 | 0.054 | 0.0194 | No |
| 15 | TNFRSF1A |  |  | 2695 | 0.050 | 0.0139 | No |
| 16 | CLIC4 |  |  | 2993 | 0.041 | -0.0143 | No |
| 17 | SERPINE1 |  |  | 3090 | 0.039 | -0.0198 | No |
| 18 | IFITM3 |  |  | 3449 | 0.029 | -0.0567 | No |
| 19 | NNMT |  |  | 3707 | 0.022 | -0.0829 | No |
| 20 | GSN |  |  | 4064 | 0.013 | -0.1219 | No |
| 21 | PLP2 |  |  | 4543 | 0.000 | -0.1767 | No |
| 22 | IGFBP7 |  |  | 5667 | -0.029 | -0.3014 | No |
| 23 | SOD2 |  |  | 5817 | -0.033 | -0.3138 | No |
| 24 | RCAN1 |  |  | 6813 | -0.072 | -0.4177 | No |
| 25 | ANXA1 |  |  | 7032 | -0.083 | -0.4308 | No |
| 26 | CLIC1 |  |  | 7041 | -0.084 | -0.4197 | No |
| 27 | WWTR1 |  |  | 7187 | -0.093 | -0.4230 | No |
| 28 | MT1X |  |  | 7275 | -0.099 | -0.4188 | No |
| 29 | ANXA2 |  |  | 8206 | -0.230 | -0.4925 | Yes |
| 30 | TAGLN2 |  |  | 8313 | -0.261 | -0.4672 | Yes |
| 31 | PRDX6 |  |  | 8410 | -0.300 | -0.4352 | Yes |
| 32 | TUBA1C |  |  | 8478 | -0.328 | -0.3958 | Yes |
| 33 | S100A11 |  |  | 8490 | -0.336 | -0.3488 | Yes |
| 34 | LGALS3 |  |  | 8522 | -0.347 | -0.3026 | Yes |
| 35 | MT2A |  |  | 8650 | -0.456 | -0.2517 | Yes |
| 36 | S100A16 |  |  | 8714 | -0.555 | -0.1792 | Yes |
| 37 | S100A10 |  |  | 8717 | -0.558 | -0.0994 | Yes |
| 38 | LGALS1 |  |  | 8744 | -0.718 | 0.0008 | Yes |
Table: GSEA details [plain text format]

  

Fig 2: RICHARDS\_NEFTEL\_CELL\_2019\_MES1: Random ES distribution      
 Gene set null distribution of ES for **RICHARDS\_NEFTEL\_CELL\_2019\_MES1**
